# Circularly Polarized Vivaldi Antennas Integrated with Septum-like Polarizer

**DOI:** 10.3390/s24134346

**Published:** 2024-07-04

**Authors:** Ilkyu Kim, Sun-Gyu Lee, Yong-Hyun Nam, Jeong-Hae Lee

**Affiliations:** 1 Department of Electrical and Electronics Engineering, Sejong Cyber University, Seoul 05000, Republic of Korea; 2 Terrestrial & Non-Terrestrial Integrated Telecommunications Research Laboratory, Electronics and Telecommunications Research Institute (ETRI), Deajeon 34129, Republic of Korea; gyul0206@etri.re.kr; 3 Department of Electronics and Electrical Engineering, Hongik University, Seoul 03967, Republic of Korea; namy129@naver.com

**Keywords:** circular polarization, Vivaldi antenna, septum polarizer, genetic algorithm, feed network design, co-planar antenna, full-wave simulation

## Abstract

In this paper, two orthogonally placed Vivaldi antennas with a septum-like polarizer to generate circular polarized (CP) waves are presented. Septum polarizers have garnered attention due to their simple structure and high quality of CP waves. While a typical septum polarizer has been applied to various types of waveguides, its applicability to the substrate integrated Vivaldi antenna is demonstrated here for the first time. A pulse train-shaped polarizer is used, which is placed on one of the two Vivaldi antennas. The contours of the polarizer are optimized using a genetic algorithm to provide an equal amplitude and 90° phase difference between the two orthogonal electric fields. In contrast to typical feed networks with a 90° phase shifter, any unwanted loss caused by an electronic circuit can be greatly mitigated. The antenna prototype was fabricated, and its radiation pattern and impedance matching were measured and compared to the simulated results.

## 1. Introduction

Circularly polarized (CP) antennas are advantageous due to their robustness against multi-path reduction, fading, and polarization mismatch between two antennas, compared to antennas with linear polarization (LP) [1,2]. A variety of antennas such as microstrip patch antennas, wire antennas, slot antennas, and horn antennas have been used for realizing CP antennas [1,3,4,5]. Among them, the Vivaldi antennas firstly proposed by P. Gibson are becoming more popular due to their high gain, broad bandwidth, and ease of impedance matching [6,7,8,9,10,11,12,13]. In recent years, there has been advancement to offer advantages of diverse polarizations employed in the Vivaldi antennas. Especially, two or more assemblies of coplanar Vivaldi antennas have been widely used to meet the requirements in terms of different types of polarization [14,15,16,17]. Dual-polarized antennas have been obtained through two co-planar LP Vivaldi antennas in an orthogonal configuration [14,15]. With the emergence of modern wireless communication, the use of CP antennas has been ever increasing. Coplanar CP Vivaldi antennas have been presented using a feed network involving a power divider to split the power equally and a 90° phase shifter [16,17]. However, within the feed network, circuitries with several electronic components are required and therefore introduce dielectric losses at high frequencies and increase the complexity of the feed network [18,19]. Therefore, an efficient CP Vivaldi antenna with the simple design of a low-loss feed network is desired.

Another alternative is a septum polarizer, which has been used in waveguide systems. A septum polarizer based on a common wall of two rectangular waveguides has been widely employed, where most studies have focused on optimizing the contour’s shape of the wall. A high quality of CP wave can be obtained using a septum polarizer, minimizing undesired losses [20,21,22,23,24,25]. A septum polarizer that has been often used in large waveguide and horn antennas has extended its applications to low profile antennas [26,27]. Some effort has been made to implement a septum polarizer for a multi-layered substrate integrated waveguide (SIW) instead of a planar antenna [26]. A septum polarizer with a low profile waveguide has been studied for use in the dual polarized antennas used in satellite communications [27]. A septum polarizer that is similar to the teeth of a wood saw has been obtained through optimizing its single tooth using a full-wave simulation tool, HFSS. This septum is advantageous due to its applicability to antennas with a low profile; however, its applications are confined to narrowband antennas, compared to the previous works.

The CP Vivaldi antenna can be used in radar and satellite communication applications to enhance the probability of target detection and communication efficiency. For some high-altitude applications, the feed network using electronic components may be affected by the severe environment in the atmosphere [28]. Therefore, a feed network with robustness against the effects of the external circumstances is more desirable.

This paper presents a circularly polarized Vivaldi antenna operating at 11 GHz incorporated with a septum polarizer, which has been frequently used in feed networks of waveguide antennae. In this work, the proposed antenna consists of vertical and horizontal Vivaldi antennas, and either is combined with the septum polarizer. A septum polarizer shaped like a pulse train is presented, which makes it possible to guide the wave until it arrives at an aperture of the antenna and produces a proper phase difference between the two orthogonal antennas. Optimizing the dimensions of the pulse train was performed using a full-wave simulation based optimization technique. In addition, in this work, non-uniform spacing between each pulse train was utilized to obtain a wide bandwidth. The prototypes of the two horizontal and vertical Vivaldi antennas were constructed and firmly attached together. The measured results are provided in order to show its validity.

## 2. Materials and Methods

### 2.1. Design Approach

**Figure 1:**
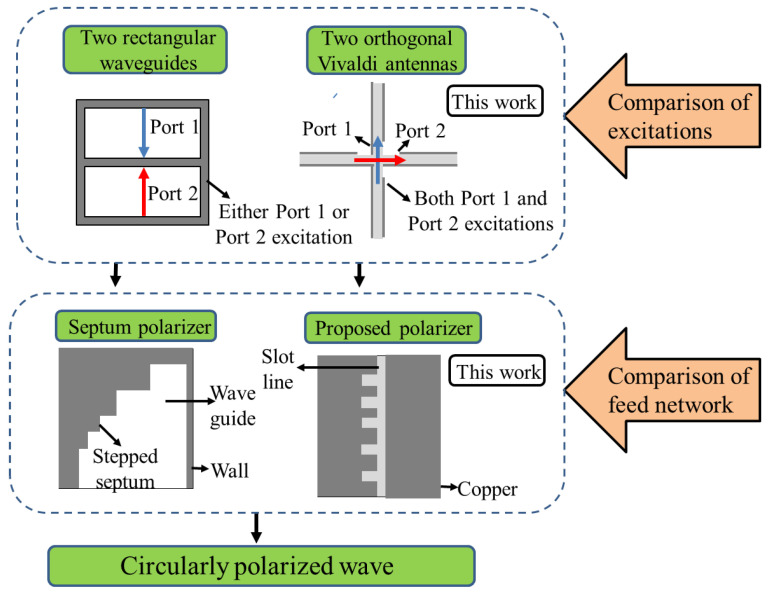
Comparison of a typical septum polarizer for conventional waveguides and a proposed polarizer for Vivaldi antennas.

Figure 1 depicts a comparison of different polarizers to generate circularly polarized waves. While the typical septum has selective excitation of either one of two ports, the proposed polarizer requires simultaneous excitations of both orthogonal ports. Although they have different configurations for port excitations, similar magnitudes and 90° phase differences can be obtained. The typical septum polarizer is placed in the transition section between the feed network and antenna aperture. However, for the Vivaldi antenna, the antenna gain is determined by the shape of the two exponential slot edges. If the proposed polarizer is situated in the radiating edges, the antenna performance may be degraded. Therefore, the proposed polarizer is located within the slotted section or feed network to avoid this adverse effect. A septum polarizer shaped like the teeth of a wood saw has been presented for a low-profile parallel plate waveguide. The proposed polarizer is different from the previous septum polarizer in terms of the shape of each element and its array configuration. A simplified pulse train is used, and the optimal spacing between each element is studied to obtain a wider bandwidth. Furthermore, the proposed polarizer is advantageous because it is more suitable for a planar antenna, which is printed on a single substrate.

### 2.2. CP Vivaldi Antenna with Ideal Dual Ports

**Figure 2:**
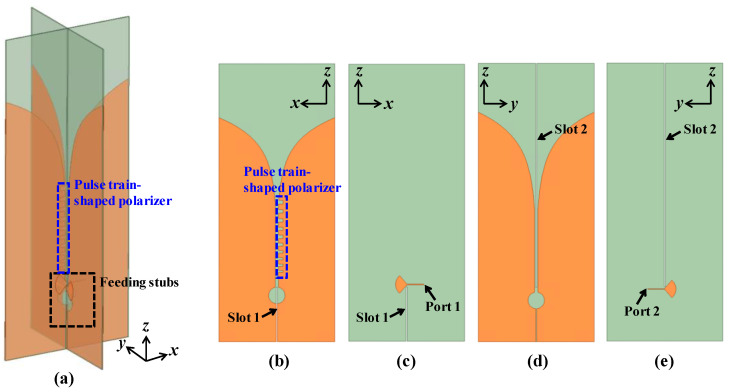
Proposed CP Vivaldi antenna with ideal two ports. (**a**) Perspective view, (**b**) top and (**c**) bottom view of the Vivaldi antenna in the *xz* plane, (**d**) top and (**e**) bottom view of the Vivaldi antenna in the *yz* plane.

**Figure 3:**
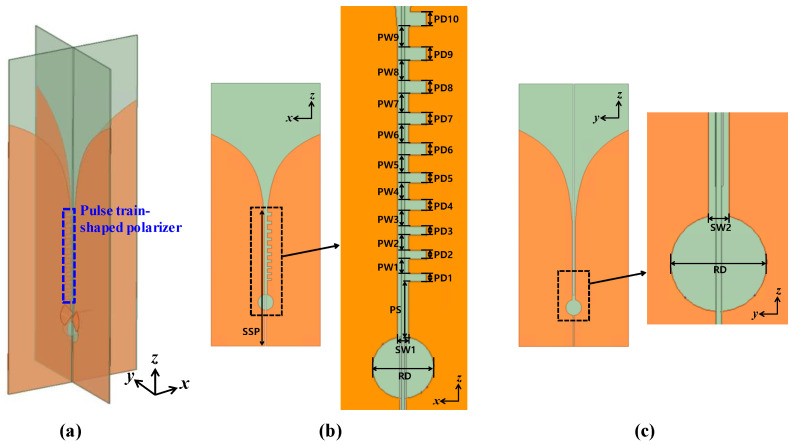
Detailed view of the proposed CP Vivaldi antenna with a pulse train-shaped polarizer. (**a**) Perspective view, (**b**) *xz* plane, and (**c**) *yz* plane. The slot edges increase exponentially, and the spacing and width of the pulse train-shaped polarizer widen in a geometric sequence for broadband AR.

To verify the design approach of the proposed CP Vivaldi antenna using a pulse train-shaped polarizer, a dual port design is considered as shown in Figure 2. The proposed CP antenna consists of two orthogonal Vivaldi antennas in the *xz* and *yz* planes, with the antenna in the *xz* plane containing a pulse train-shaped polarizer whose width is widened in a geometric sequence. As the phase difference between the dual ports is set to 0° or 180°, the CP Vivaldi antenna operates in a right-handed circular polarization (RHCP) or left-handed circular polarization (LHCP) mode, respectively. The impedance of the dual ports and feeding lines is set to 100 Ω considering the connection with the feeding network in the next step. In addition, the dielectric slots exist for orthogonal physical assembly. For broadband impedance matching, the feeding structure of the open-ended tapered micro-strip lines (or feeding radial stubs) and circular cavity are adopted, the same as for conventional CP Vivaldi antennas. Two orthogonal Vivaldi antennas are implemented as exponential slot edges for broadband impedance matching, and the shape of the slot edges is determined from the following formula:

(1) $$x=\mathrm{SSP}+\frac{{\mathrm{SW}}_{m}}{2}\times e^{\varphi_{m}z}$$

where SSP, SW*_m_*, and φ*_m_* represent the starting point, width, and tapering angle of the slot. *m* = 1 and *m* = 2 represent the cases for the *xz* and *yz* planes, respectively. Also, RD is set to be 10 mm. The detailed structure of the CP Vivaldi in the *xz* and *yz* planes is shown in Figure 3. The pulse train-shaped polarizer is designed with increasing pulse spacing (PD*_n_*) and width (PW*_n_*) for broadband impedance matching, where *n* represents the stage index of the pulse train. The total number of stages in the pulse trains is set to be nine for the wide axial ratio (AR) bandwidth. As the pulse train-shaped polarizer is added, the impedance mismatch occurs for the Vivaldi antenna in the *xz* plane, causing an imbalance in the radiation intensity of the two orthogonal antennas. Thus, by optimizing the slot width (SW*_m_*) and the shape of the exponential slot edge in the *xz* and *yz* planes, the radiation intensity of the two orthogonal polarizations can be balanced. To reduce the number of optimization cases, the PD*_n_* and PW*_n_* of each stage are defined by the following geometric sequence models:

(2) $${\mathrm{PD}}_{n+1}={\mathrm{PD}}_{n}+{\mathrm{PD}}_{\mathrm{add}}$$

(3) $${\mathrm{PW}}_{n+1}={\mathrm{PW}}_{n}+{\mathrm{PW}}_{\mathrm{add}}$$

where the PD_1_ and PW_1_ correspond to the initial pulse distance and width, and PD_add_ and PW_add_ correspond to the common radio of the geometric sequence. Finally, ten variables of PS, PD_1_, PW_1_, PD_add_, PW_add_, SSP, SW_1_, SW_2_, φ_1_, and φ_2_ are specified that mainly determine the performance of the proposed CP Vivaldi antenna. However, the performance optimization faces the challenge of being time-consuming due to the multiple variables.

### 2.3. Performance Optimization Using Genetic Algorithm

**Figure 4:**
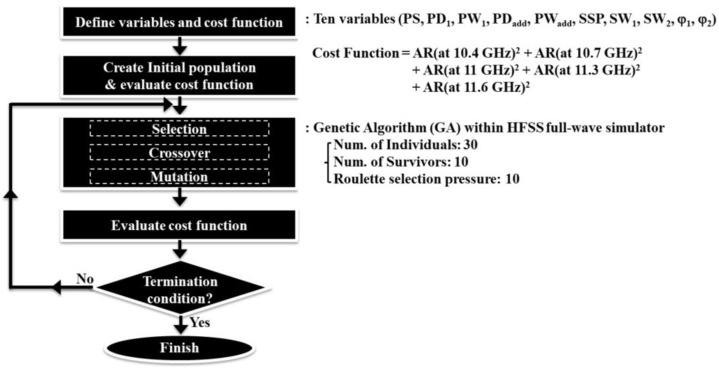
Schematic of the GA optimization process.

To easily optimize the performance of the proposed CP Vivaldi antenna, a genetic algorithm (GA) within the HFSS full-wave simulation is used. Figure 4 schematizes the entire process of GA optimization. Firstly, we designate the parameters that most affect its performance as variables. Ten variables are specified for the optimization: PS, PD_1_, PW_1_, PD_add_, PW_add_, SSP, SW_1_, SW_2_, φ_1_, and φ_2_. The center frequency of the proposed CP Vivaldi antenna is set to be 11 GHz, and the GA cost function is defined as follows with the AR values in the range 10.4–11.6 GHz at 300 MHz intervals:

(4) $$\mathrm{Cost}\;\mathrm{Function}=\mathrm{AR}{\left(\mathrm{at}\;10.4\;\mathrm{GHz}\right)}^{2}+\mathrm{AR}{\left(\mathrm{at}\;10.7\;\mathrm{GHz}\right)}^{2}+\mathrm{AR}{\left(\mathrm{at}\;11\;\mathrm{GHz}\right)}^{2}+\mathrm{AR}{\left(\mathrm{at}\;11.3\;\mathrm{GHz}\right)}^{2}\;+\mathrm{AR}{\left(\mathrm{at}\;11.6\;\mathrm{GHz}\right)}^{2}.$$

And then, we create the initial population and evaluate the new cost function. Next, we evaluate the cost function by the population created through the process of selection, crossover, and mutation. If the new cost function is greater than the cost function by the previous population, repeat the GA loop. If the new cost function satisfies the termination condition (i.e., the cost function is close to 0), finish the GA optimization. The GA settings within HFSS are specified as follows: number of individuals = 30, number of survivors = 10, and Roulette selection pressure = 10. Because Vivaldi antennas already have secured broadband impedance matching, only ARs are included in the cost function. The optimal design corresponds to the case with the smallest cost function value. After performing approximately 2200 simulations, the lowest cost function value was obtained as 36, which is considered the optimal design. Table 1 lists the parameters of the optimized CP Vivaldi antenna.

**Figure 5:**
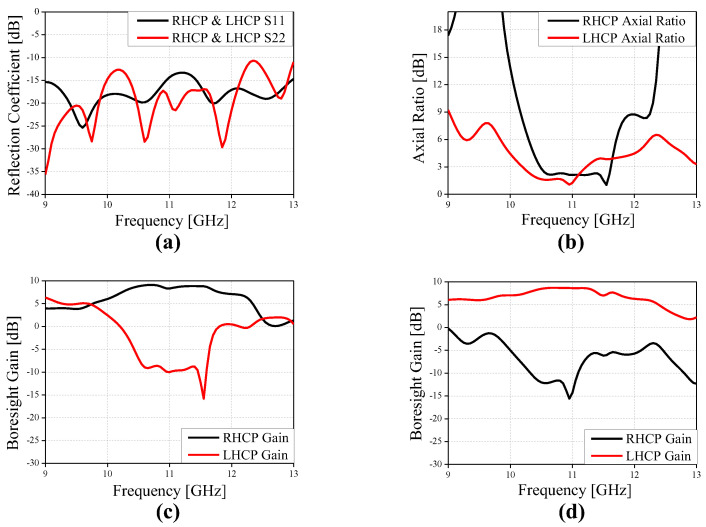
Performance of the proposed CP Vivaldi antenna with ideal two ports. (**a**) Reflection coefficient, (**b**) axial ratio, (**c**) boresight gain for RHCP, and (**d**) boresight gain for LHCP.

**Figure 6:**
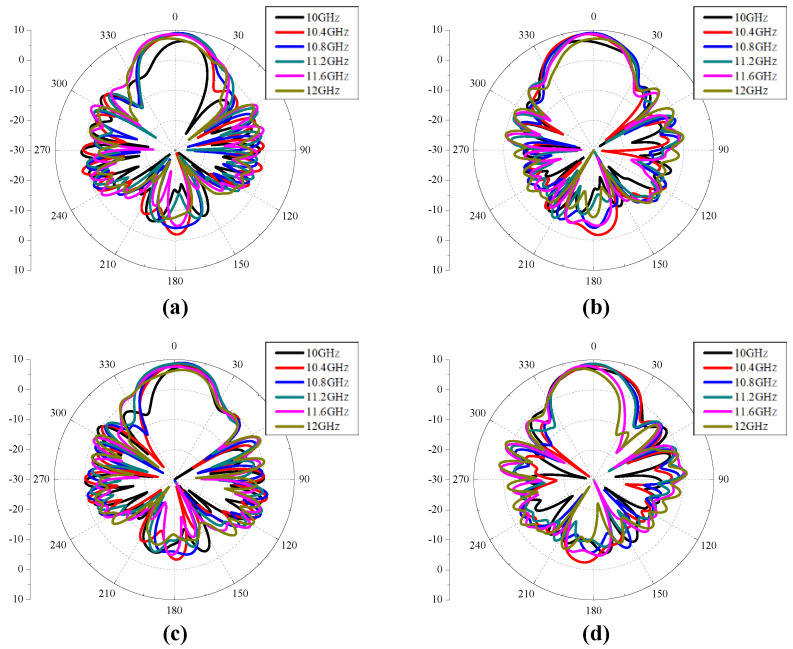
Co-polarized radiation patterns of the proposed CP Vivaldi antenna with ideal two ports: (**a**) RHCP mode & *xz* plane, (**b**) RHCP mode & *yz* plane, (**c**) LHCP mode & *xz* plane, and (**d**) LHCP mode & *yz* plane.

Figure 5 shows the reflection coefficient, AR, co- and cross-polarized gain of a CP Vivaldi with dual ports. The reflection coefficients S_11_ and S_22_ are maintained below −10 dB over a wide bandwidth. The 3 dB AR bandwidth is 1.14 GHz (=10.3%) for RHCP and 1.03 GHz (=9.6%) for LHCP, the peak gain is 9.1 dB for RHCP and 8.7 dB for LHCP, and the −1 dB gain bandwidth is 1.35 GHz (=12.3%) for RHCP and 1.1 GHz (=10.1%) for LHCP, respectively. In addition, flat gain characteristics are observed in a wide band. The stable directional radiation pattern for 10.4–11.6 GHz is also confirmed as shown in Figure 6. Therefore, the design concept of the proposed CP Vivaldi antenna with a pulse train-shaped polarizer is verified for operation in RHCP and LHCP modes in broadband.

### 2.4. CP Vivaldi Antenna with Feeding Network

**Figure 7:**
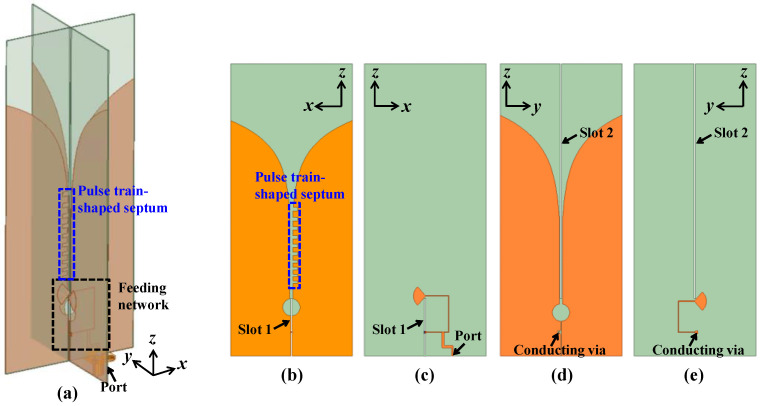
Proposed CP Vivaldi antenna combined with feeding network. (**a**) Perspective view, (**b**) top and (**c**) bottom view of the Vivaldi antenna in the *xz* plane, (**d**) top and (**e**) bottom view of the Vivaldi antenna in the *yz* plane.

In practical applications, a single port design is preferred for system compatibility. The CP Vivaldi antenna with a single port can be easily implemented by adding a simple T-junction power divider. Figure 7 shows the structure of the proposed CP Vivaldi antenna with a single port combined feeding network instead of a dual port. Note that, except for the feeding network, the other structures are the same as in Figure 2 and Figure 3.

**Figure 8:**
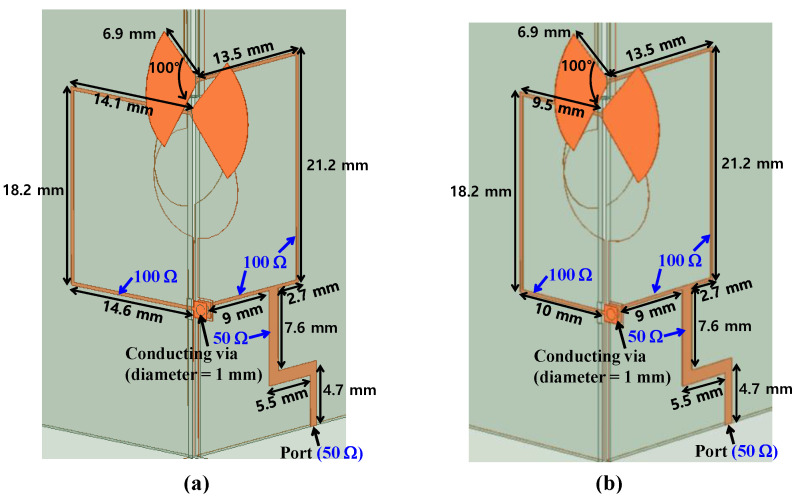
Structures of feeding networks for (**a**) RHCP and (**b**) LHCP designs.

Figure 8 shows the detailed structure of the feeding network. The 50 Ω port is connected through a microstrip line with a characteristic impedance of 50 Ω to two microstrip lines with a characteristic impedance of 100 Ω, and finally to the feeding position of the Vivaldi antennas with a local impedance of 100 Ω. A conducting via with a diameter of 1 mm is used to connect two orthogonal planes, where the influence of the conducting viaduct can be neglected due to the short path. Meanwhile, operation modes with RHCP or LHCP can be selected by setting the path difference of the left and right microstrip lines to be in-phase or out-of-phase. Figure 8a,b show the design for both RHCP and LHCP designs. The path length of the right microstrip line with a characteristic impedance of 100 Ω is kept at 37.4 mm (=2λ_g_), while the path length of the left microstrip line with a characteristic impedance of 100 Ω is specified to 56.9 mm (=3λ_g_) and 46.7 mm (=2.5λ_g_) for the RHCP and LHCP designs, respectively.

**Figure 9:**
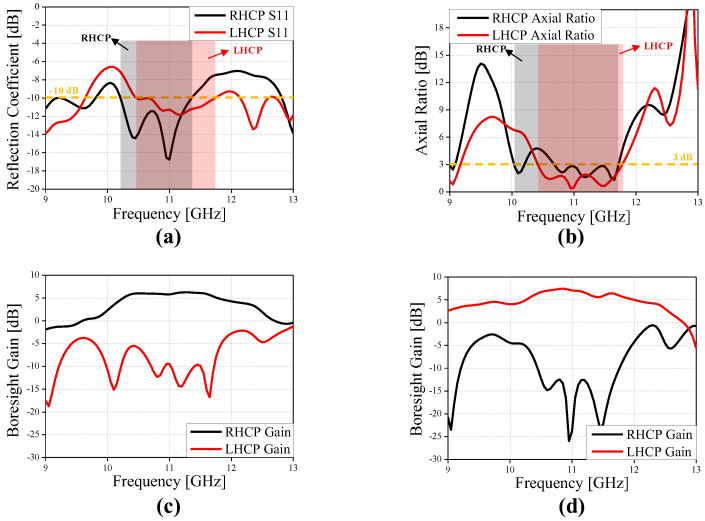
Performance of the proposed CP Vivaldi antenna: (**a**) reflection coefficient, (**b**) axial ratio, (**c**) boresight gain for RHCP, and (**d**) boresight gain for LHCP.

**Figure 10:**
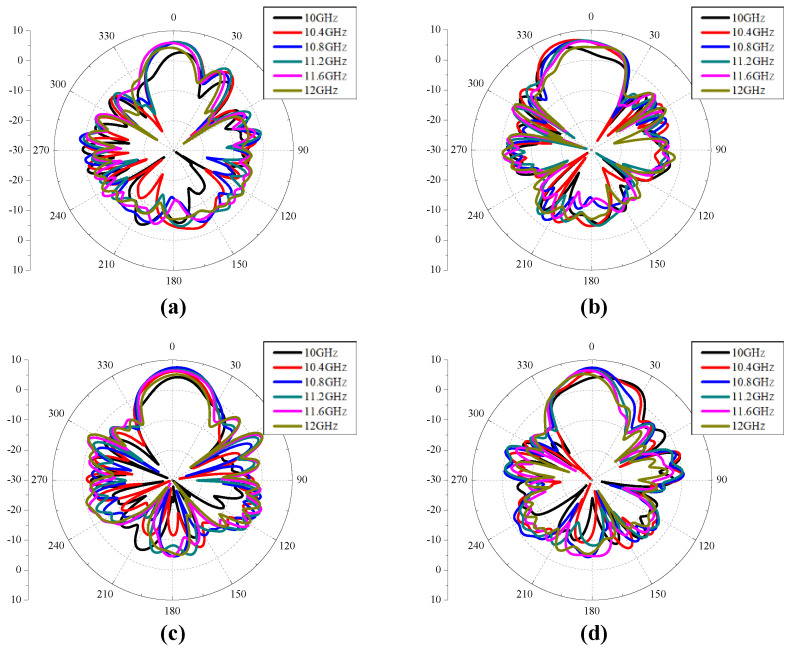
Co-polarized radiation patterns for the peak gain of the proposed CP Vivaldi antenna with feeding network: (**a**) LHCP mode and *xz* plane, (**b**) LHCP mode and *yz* plane, (**c**) RHCP mode and *xz* plane, and (**d**) RHCP mode and *yz* plane.

Figure 9 and Figure 10 shows the reflection coefficient, AR, co- and cross-polarized gain, and radiation pattern of the CP Vivaldi with the feeding network. The simulated −10 dB bandwidth of the reflection coefficients S_11_ is 1.15 GHz (=10.7%) for RHCP and 1.27 GHz (=11.4%) for LHCP, the simulated 3 dB AR bandwidth is 1.03 GHz (=9.2%) for RHCP and 1.36 GHz (=12.3%) for LHCP, the simulated peak gain is 6.2 dB for RHCP and 7.5 dB for LHCP, and the simulated −1 dB gain bandwidth is 1.46 GHz (=13.2%) for RHCP and 0.78 GHz (=7.2%) for LHCP, respectively. The discrepancies from the results of the CP Vivaldi antenna with dual ports are mainly due to the asymmetry of the T-junction power divider, microstrip line losses, and the curved microstrip line paths, etc.

## 3. Experimental Verification

**Figure 11:**
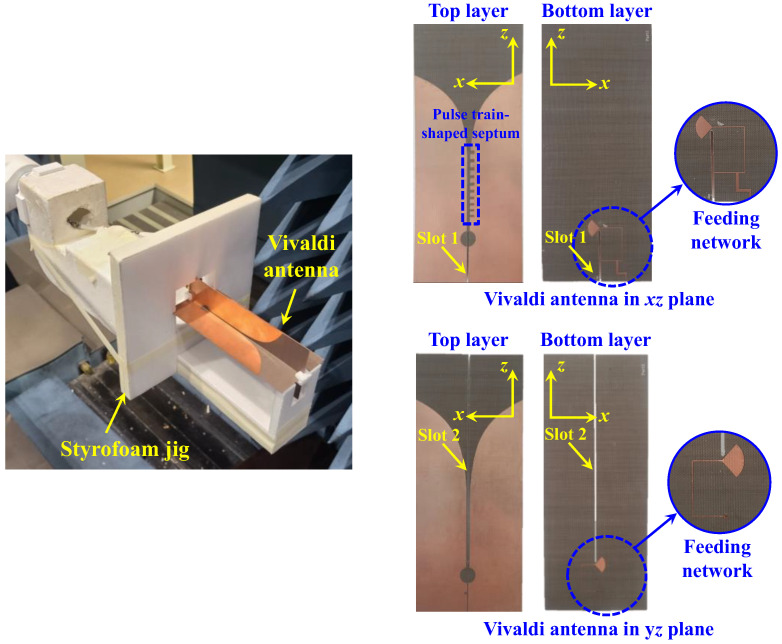
Prototype of LHCP Vivaldi antenna.

For verification of the CP Vivaldi antenna designed in full-wave simulation, fabrication and measurements were performed on the structure as shown in Figure 11. Because the −10 dB reflection coefficient and 3 dB AR bandwidth of the LHCP design are more stable than the RHCP design, the LHCP Vivaldi antenna was fabricated as a prototype for experimental verification. Anritsu’s MS46522B vector network analyzer and an anechoic chamber containing Keysight’s E8362B network analyzer was used to measure the reflection coefficient and radiation patterns. The receiving antenna was positioned 7 m away from the fabricated prototype. The fabricated LHCP Vivaldi antenna was mounted on a Styrofoam jig for orthogonal coupling.

**Figure 12:**
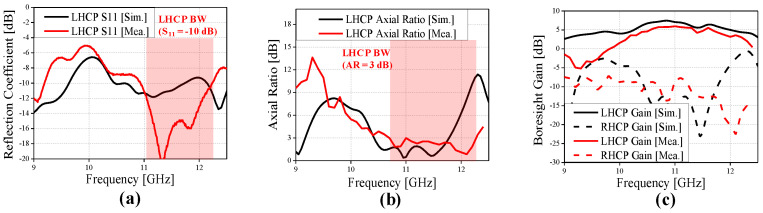
Performance of the proposed CP Vivaldi antenna: (**a**) reflection coefficient, (**b**) axial ratio, (**c**) boresight gain for RHCP design.

**Figure 13:**
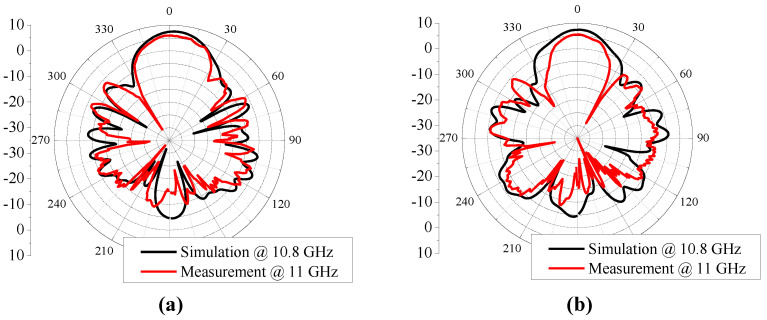
Co-polarized radiation patterns for the peak gain of the proposed CP Vivaldi antenna with feeding network: (**a**) LHCP mode and *xz* plane, (**b**) LHCP mode and *yz* plane.

Figure 12 and Figure 13 show the measurement results of the fabricated prototype. The measured results were confirmed to have a bandwidth of reflection coefficient S_11_ of 1.29 GHz (=9.7%), 3 dB AR of 1.57 GHz (=13.7%), peak gain of 6 dB, and −1 dB gain bandwidth of 0.68 GHz (=6.2%), respectively. Compared to the results of the simulation, the bandwidths of S11 and peak gain were narrowed by 1.7 dB and 1 dB, the 3 dB AR was widened by 1.4 dB, and the peak gain was reduced by 1 dB, respectively. Also, it is confirmed that the center frequencies are upshifted by approximately 0.2 GHz compared to the simulated results. The differences between the simulated and measured results is mainly due to fabrication errors in the width of the dielectric slot and microstrip lines, imperfect hand soldering for assembly, and bending of the substrate during the fabrication process, etc. Nevertheless, the simulated and measured results were found to have similar trends.

## 4. Discussion

A septum-like polarizer shaped like a pulse train has been presented for a circularly polarized Vivaldi antenna. The profile of the polarizer has been optimized using GA based on the commercial software of HFSS. The optimized design provides more than 10% fractional bandwidth in terms of a 3 dB axial ratio. Within the bandwidth, a good impedance matching was obtained. The requirements for the antenna bandwidth can be met for the use of radar and satellite communication systems. The prototype of the proposed antenna was constructed, and the simulated results showed good agreement with the experimental results. The length of the feed line can be adjusted to change the polarization between RHCP and LHCP. The design of the proposed antenna can be scalable to other frequencies under 11 GHz. However, the scalability to other frequencies above 11 GHz may be limited because the substrate becomes thinner and the rigidness of the antenna prototype may be affected. In the future, some switches to reconfigure the feed network may be added to realize a switchable CP Vivaldi antenna.

## Figures and Tables

**Table 1:** Optimal parameters through using the genetic algorithm of the full-wave simulation tool, HFSS.

Parameters	Dimension	Parameters	Dimension
PS	9 mm	SSP	85 mm
PD_1_	1.29 mm	SW_1_	1.9 mm
PW_1_	2.34 mm	SW_2_	2.09 mm
PD_add_	0.11 mm	φ_1_	60°
PW_add_	0.13 mm	φ_2_	68.9°

## Data Availability

The datasets presented in this article are not readily available because the data are part of an ongoing study.
